# Reduced Protumorigenic Tumor-Associated Macrophages With Statin Use in Premalignant Human Lung Adenocarcinoma

**DOI:** 10.1093/jncics/pkz101

**Published:** 2019-12-13

**Authors:** Esraa Al Dujaily, Juvenal Baena, Madhumita Das, Marco Sereno, Claire Smith, Tamihiro Kamata, Leah Officer, Catrin Pritchard, John Le Quesne

**Affiliations:** 1 Leicester Cancer Research Centre, University of Leicester, Leicester Royal Infirmary, Leicester, UK; 2 MRC Toxicology Unit, Leicester, UK

## Abstract

**Background:**

Statins have anticancer properties by acting as competitive inhibitors of the mevalonate pathway. They also have anti-inflammatory activity, but their role in suppressing inflammation in a cancer context has not been investigated to date.

**Methods:**

We have analyzed the relationship between statin use and tumor-associated macrophages (TAMs) in a cohort of 262 surgically resected primary human lung adenocarcinomas. TAMs were evaluated by multiplex immunostaining for the CD68 pan-TAM marker and the CD163 protumorigenic TAM marker followed by digital slide scanning and partially automated quantitation. Links between statin use and tumor stage, virulence, and cancer-specific survival were also investigated in a wider cohort of 958 lung adenocarcinoma cases. All statistical tests were two-sided.

**Results:**

We found a statin dose-dependent reduction in protumorigenic TAMs (CD68+CD163+) in both stromal (*P* = .021) and parenchymal (*P* = .003) compartments within regions of in situ tumor growth, but this association was lost in invasive regions. No statistically significant relationship between statin use and tumor stage was observed, but there was a statin dose-dependent shift towards lower histological grade as assessed by growth pattern (*P* = .028). However, statin use was a predictor of slightly worse cancer-specific survival (*P* = .032), even after accounting for prognostic variables in a multivariable Cox proportional hazards survival model (hazard ratio = 1.38, 95% confidence interval = 1.04 to 1.84).

**Conclusions:**

Statin use is associated with reduced numbers of protumorigenic TAMs within preinvasive lung adenocarcinoma and is related to reduced tumor invasiveness, suggesting a chemo-preventive effect in early tumor development. However, invasive disease is resistant to these effects, and no beneficial relationship between statin use and patient outcome is observed.

Statins are a class of safe and well-tolerated cholesterol-lowering drugs commonly used for cardio-protective benefits. They are one of the most prescribed medications, and thus there has been interest in their relationship with malignancy. However, the link between statin use and cancer incidence and mortality is controversial, with some studies showing a relationship between lower cancer incidence (1–3) or reduced cancer mortality (4,5) and other studies not supporting these conclusions (6–8).

Despite a lack of clear correlation between statin use and outcomes for patients, there is abundant preclinical evidence to support their anticancer functions (9,10). The best characterized mechanism through which statins exert their anticancer effect is through their ability to suppress the mevalonate pathway by inhibition of 3-hydroxy-3-methyl-glutaryl-CoA reductase, leading to reduced cholesterol production and protein prenylation, both of which are vital for cancer cell proliferation and survival (9,11). Statins also have anti-inflammatory functions independent of their ability to lower cholesterol (12). This is achieved through a variety of mechanisms, including suppression of cytokine and cell adhesion molecule expression (13–15) and inhibition of NF-κB signalling (13,16,17).

Cancer development is dependent on an interaction between tumor cells and the microenvironment (18) with inflammation being a key factor in this (19). Monocytes or macrophages are important contributors to chronic inflammation through their plasticity and capacity to become polarized by the tumor microenvironment to form tumor-associated macrophages (TAMs) (20,21). The two main cancer-relevant TAM subtypes are the M2-polarized immunosuppressive prooncogenic and M1-polarized tumoricidal antioncogenic forms (20,22). A link between higher prooncogenic TAM number in the tumor stroma and worse clinical prognosis has been demonstrated for several cancers, including lung cancers (23–25). Studies in animal models support an association between TAMs and enhanced tumor progression (26), and depleting TAMs by splenectomy results in regression of oncogenic KRAS-driven mouse lung premalignant and advanced lesions (27).

In searching for therapies that may counteract the protumorigenic activities of TAMs, statins have been considered a possibility. The aim of this study was to investigate the impact of statin use on TAM location, number, and polarity and correlate these with analysis of tumor proliferation, stage, grade, and survival in a cohort of lung adenocarcinoma samples from patients undergoing surgical resections with curative intent.

## Methods

### Patient Cohort

A continuous cohort of 958 primary lung adenocarcinomas resected with curative intent between the years 1998 and 2015 was identified from the University Hospitals of Leicester NHS Trust histopathology database under a project protocol supported by the regional ethics committee (ref 157104/UHL11363) and made use of the consent exemption detailed in the UK Human Tissue Act 2004 (www.hta.gov.uk). Cases were excluded if there was a possibility of nonprimary lung origin or synchronous primary pulmonary nonadenocarcinoma. All patients were treatment naïve at the time of surgery. All key prognostic variables were compiled from pathology databases and from patient notes, including patient sex, age, performance status, and key determinants of stage such as tumor size and nodal involvement at the time of surgery. The involvement of pleura, presence or absence of vascular invasion, and predominant growth pattern was confirmed by dual examination of diagnostic slide images by two histopathologists (J. Le Quesne and J. Baena). Drug history data were obtained from hospital databases and patient notes. Statin use status was defined at the time of admission for surgery, and duration of statin use was calculated as the date of treatment initiation to the date of surgery.

### Tissue Microarrays

A subset of 262 resected primary lung adenocarcinoma samples from 2012 to 2014 was assembled into tissue microarrays (TMAs). Scanned images of all diagnostic blocks were examined, and regions were selected for inclusion into TMAs. In cases of mixed histological growth pattern, most cores representing diverse growth patterns were sampled, with the sampling of both in situ and invasive areas being the priority. Five TMA blocks containing 3×1-mm cores per case were constructed using a semiautomatic array machine TMArrayer (Pathology Device, San Diego, California, USA). We cut 5-µm sections for subsequent staining and analysis.

### Immunohistochemistry

Slides were dewaxed by placing in 100% xylene for 10 minutes each. They were rehydrated through graded alcohols (2 × 1 minute in 99% [v/v] Industrial Methylated Spirits (IMS) followed by 1 minute in 95% [v/v] IMS) and then placed in running tap water for 5 minutes. Antigen retrieval was performed by placing slides in citrate buffer (0.01 M citric acid, pH 6.0) and microwaving at full power for 20 minutes. Peroxidase blocking solution, supplied with the Polymer detection system (Leica Biosystems, Wetzlar, Germany; RE7230-K and RE7270-K), was applied to the slides for 5 minutes to neutralize endogenous peroxidases. After washing in Tris-buffered saline (TBS), slides were placed in a protein block solution supplied with the polymer detection system kit for 5 minutes and then washed twice in TBS for 5 minutes each. The slides were incubated for 1 hour at room temperature either with a single antibody or cocktail of primary antibodies using appropriate dilutions. Slides were washed in TBS for 5 minutes before applying the appropriate secondary antibodies. Primary antibodies used were a 1:400 dilution of a CD68 rabbit polyclonal antibody (Sigma-Aldridge (Merck; St Louis, Missouri USA), clone HPA048982), a 1:600 dilution of a CD163 mouse monoclonal antibody (Novocastra (Leica Biosystems), clone 10D6), and a 1:100 dilution of a Ki67 antibody (Abcam (Cambridge, UK) AB15580). For double immunohistochemistry, the dual staining system from MenaPath (A.Menarini Diagnostics, Wokingham, UK) was used (MP-XLCT525-K6) according to the manufacturer's instructions. After staining, slides were counterstained by incubation in Mayer’s hematoxylin for 30 seconds before dehydration and mounting.

### Digital Scanning and Image Analysis

Stained slides were scanned using a Hamamatsu scanner NanoZoomer-XR Digital slide scanner (Hamamatsu Photonics UK Ltd, Welwyn Garden City, UK), and images were analyzed using Visiopharm software (Horsholm, Denmark). Luminal, stromal, and tumor areas were separated manually in Visiopharm on the counterstained immunohistochmical (IHC) images, with reference to hemotoxylin and eosin (H&E)-stained serial sections where necessary. Cores in which microanatomical region assignments were difficult were further examined by a subspecialty respiratory pathologist (J. Le Quesne) and consensus segmentation was applied. Tumor islands or parenchyma, stroma, and luminal compartments were analyzed separately. An app was designed using Visiopharm software to identify and count all macrophages (CD68-positive) and protumorigenic polarized macrophages (dual positivity for CD68 and CD163). Individual cores were categorized as in situ or invasive by two pathologists (J. Baena and J. Le Quesne) working in a blinded manner, with discussion and consensus assignment of equivocal cores. In situ cores contained only in situ or lepidic growth pattern malignancy, with cytologically malignant cells growing on the surface of architecturally normal lung alveoli. “Invasive” cores contained invasive growth pattern morphology (acinar, papillary, solid, and micropapillary) as defined by World Health Organization diagnostic criteria.

### Statistical Analysis

Associations between continuous variables were assessed by Spearman rank correlation. Associations between binary variables and between binary and ordinal variables were assessed with the Pearson χ^2^ test. Trends in continuous variables across ordered groups were assessed using the Cuzick test for trend. Differences in continuous variables between unpaired groups were assessed using the Mann-Whitney *U* test. Stata SE 15.1 was used for all statistical figure preparation and statistical and survival analyses.

### Survival Analysis

Time-to-event data on cancer-specific survival status and time from surgery to date of last-known status were obtained from the cohort database using a follow-up time of 6 years. The cancer-specific survival endpoint used as events only deaths directly attributable to lung cancer (ie, within part I of the UK medical certificate of cause of death) and non-lung cancer deaths were censored. Survival was modeled using Kaplan-Meier analysis, and differences between groups were assessed by the log-rank test. A multivariable Cox proportional hazards model was built to incorporate all available additional known prognostic variables. The proportional hazards assumption was tested by examination of log-log plots. All tests were two-sided and a P value of less than .05 was considered statistically significant.

## Results

**Figure 1.**
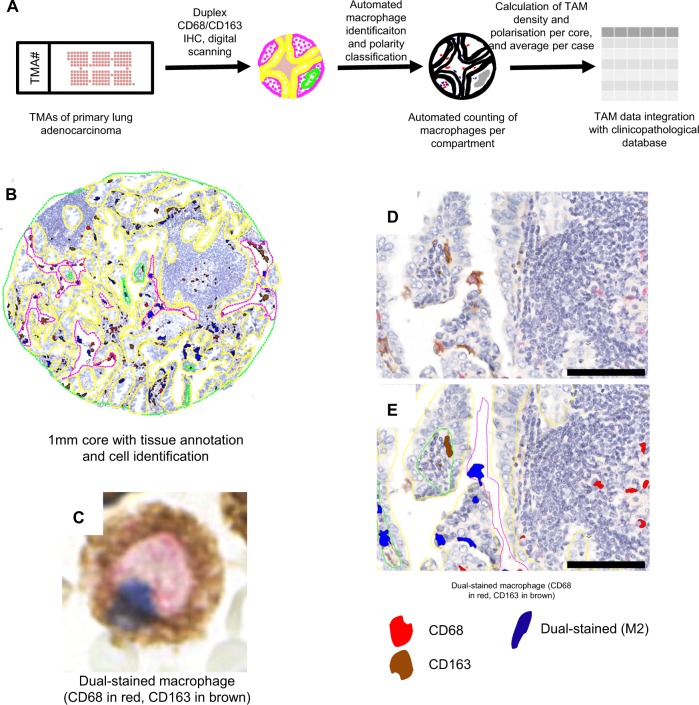
In situ macrophage density and polarity analysis. **A**) Automated macrophage polarity detection and quantification. **B**) A low-power view of a representative 1-mm core of invasive human pulmonary adenocarcinoma with superimposed manual segmentation into epithelial or stromal (**yellow**), luminal (**pink**), and necrotic (**green**) areas. **C**) High-power view of a dual-stained protumorigenic macrophage. **D**) Medium-power view showing multiple macrophages with diverse staining patterns. **E**) Medium-power view with superimposed automated cell classifications. IHC = immunohistochemical; TAM = tumor-associated macrophage; TMA = tissue microarray.

We first assessed the distribution of TAMs within lung adenocarcinoma samples by multiplex analysis of TMAs constructed from a cohort of 262 lung adenocarcinoma samples, the details of which are provided in Table 1. TMAs were immunostained with antibodies for the pan-macrophage marker CD68 and the putative prooncogenic macrophage marker CD163. Macrophages were identified and classified algorithmically in digital images, and subclasses of macrophages were counted in parenchymal, stromal, and luminal tumor compartments. The staining patterns were analyzed by digital scanning and quantitation (Figure 1).

**Table 1.** Statin use in lung adenocarcinoma cohort in tissue microarrays

Variable	No. (%)
Statin use	
No statin	149 (56.9)
Statin	112 (42.8)
Unknown	1 (0.4)
Statin type	
Atorvastatin	29 (25.9)
Simvastatin	75 (67.0)
Other	8 (7.1)
Statin dose	
<40 mg	51 (45.5)
≥40 mg	61 (54.5)
Statin duration	
<1 mo	39 (34.8)
1–6 mo	35 (31.3)
>6 mo	36 (32.1)
Unknown	2 (2.7)

**Figure 2.**
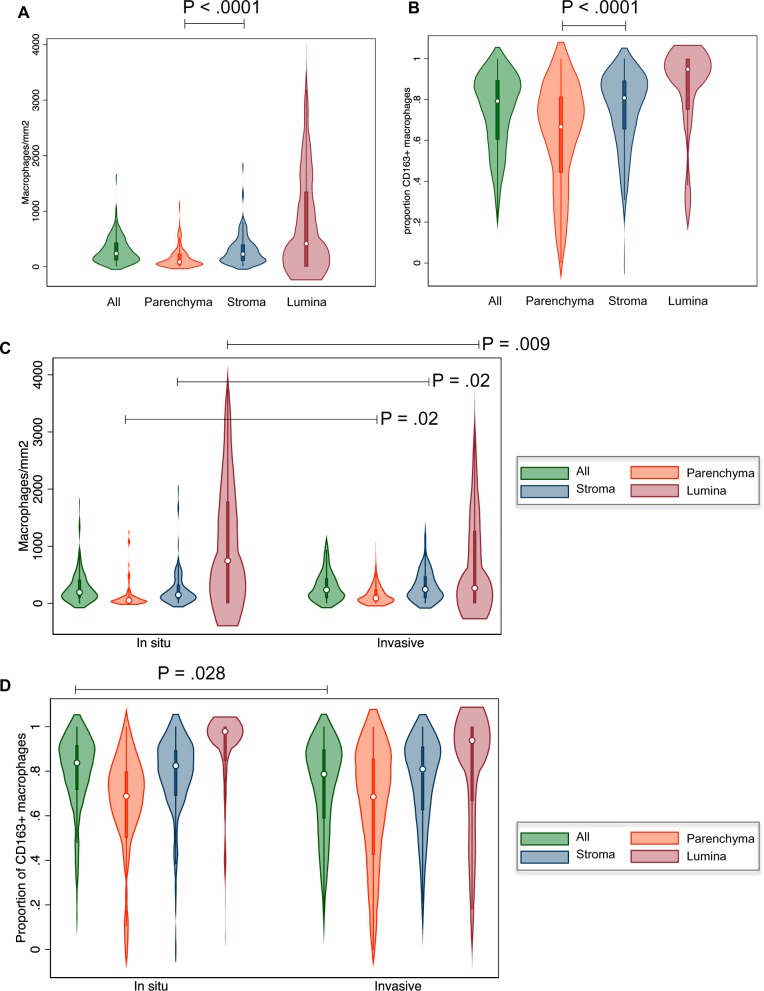
Compartmentalized macrophage number and polarity. **A**) Quantitation of CD68+ tumor-associated macrophages (TAMs) in the tumor parenchyma, stroma, and lumina. **B**) Quantitation of CD163+/CD68+ TAMs in the tumor parenchyma, stroma, and lumina. **C**) Quantitation of CD68+ TAMs overall and in the tumor parenchyma, stroma, and lumina subcompartments of in situ and invasive regions. **D**) Quantitation of CD163+/CD68+ TAMs overall and in the tumor parenchyma, stroma, and lumina subcompartments of in situ and invasive regions. These data were established by immunohistochemical staining of the tissue microarrays (TMAs) harboring 262 lung adenocarcinoma samples with antibodies for CD68 and CD163 followed by automated cell classification and counting. Numbers were normalized to the areas of manually drawn tissue compartments.

The density of total CD68+ TAMs (Figure 2A) and the proportion of CD68+/CD163+ TAMs (Figure 2B) were both statistically significantly lower in parenchymal (ie, epithelial) tumor areas compared with tumor stroma. Individual tissue cores were classified as being of either in situ or invasive pattern, and we found that TAM densities were statistically significantly higher in invasive areas compared with in situ areas in both parenchymal and stromal zones (Figure 2C). In luminal areas, the reverse relationship was seen in that there were fewer luminal macrophages within invasive tumor areas. This perhaps reflects the physiological differences between lumina within in situ disease, which are continuous with healthy airways that have their own pulmonary alveolar macrophage populations, compared with lumina, which arise de novo in disordered areas of invasive tumor growth. Invasive areas showed a mild elevation of protumorigenic polarization overall (*P* = .028), although this was not observed when analyzed by microanatomical subcompartment (Figure 2D).

**Figure 3.**
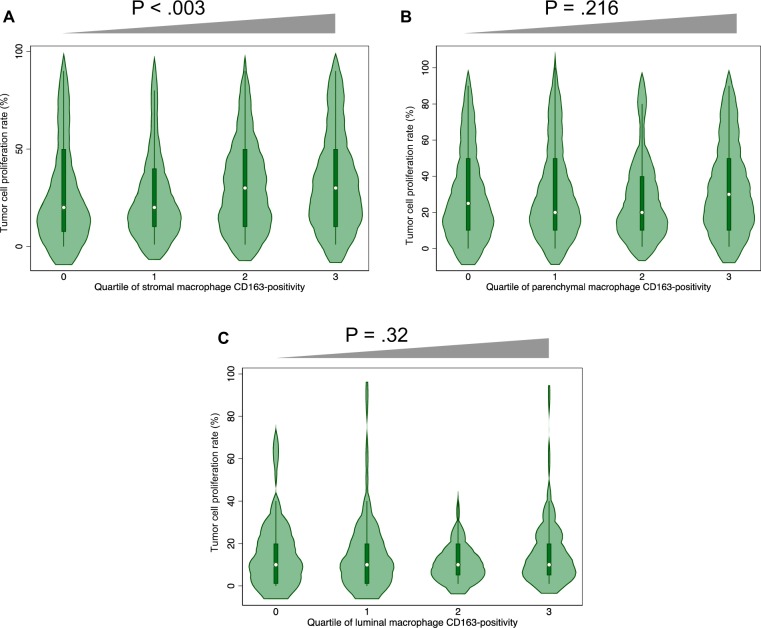
Relationship between macrophage polarity and tumor cell proliferation. Tissue microarrays (TMAs) were immunostained with antibodies for Ki67, CD163, and CD68 followed by automated quantification of the stained cells in stromal (**A**), parenchymal (**B**), and luminal (**C**) areas. The **gray triangles** above the figures indicate results of tests for trend across ordered categories; only stromal macrophages are related to tumor cell proliferation.

We investigated the link between TAMs and tumor proliferation and found a strong positive association between the density of CD68+/CD163+ TAMs in the stroma and proliferation of tumor cells as determined by Ki67 staining (Figure 3A), but no such association was detected in parenchymal (Figure 3B) or luminal (Figure 3C) areas. Protumorigenic TAMs such as those that can be identified by dual staining with CD68 and CD163 are known to promote tumor cell proliferation, angiogenesis, invasion, and metastasis as well as to suppress T-cell–mediated antitumor immune responses through paracrine mechanisms (20,24,25). Our finding of an association of CD68+/CD163+ TAMs with increased tumor cell proliferation is consistent with this protumorigenic function and argues in favor of a direct mitogenic, paracrine effect rather than a reduction in immune-mediated cytotoxicity.

**Figure 4.**
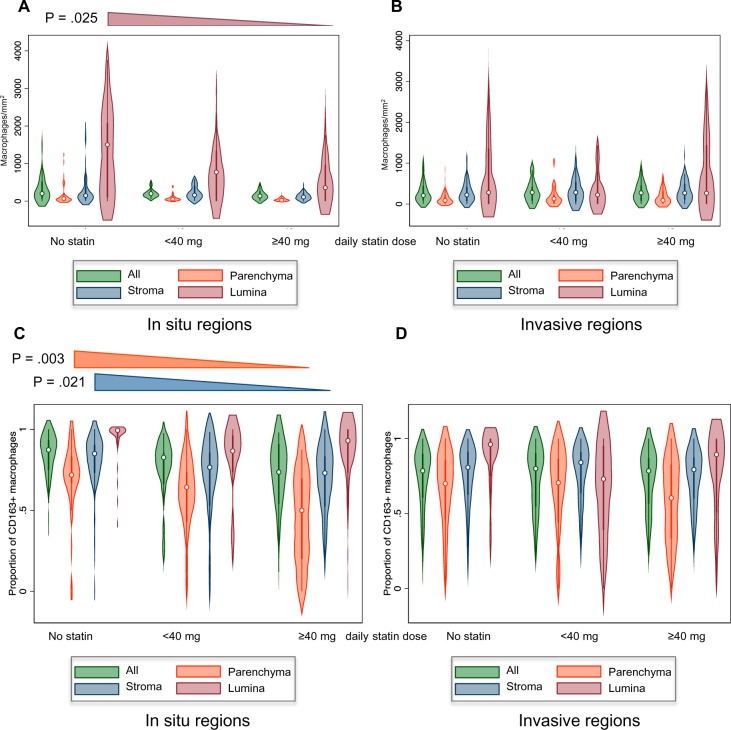
Relationship between statin use and tumor-associated macrophage (TAM) number and polarity within in situ and invasive regions of lung adenocarcinoma samples. **A**) Quantitation of CD68+ TAMs overall and in microanatomical compartments of in situ tumor regions from statin- and nonstatin-treated patients. The **extended triangle** above the figure indicates a statistically significant trend within luminal macrophages. All other categories were not statistically significant. **B**) Quantitation of CD68+ TAMs in microanatomical compartments of invasive tumor regions. No statistically significant trends were observed. **C**) Quantitation of the fraction of prooncogenic CD68+/CD163+ TAMs in microanatomical compartments of in situ tumor regions. The **extended triangles** above the figure indicate statistically significant trends across parenchymal macrophages (**orange**) and stromal macrophages (**blue**). All other categories were not statistically significant. **D**) Quantitation of the fraction of prooncogenic CD68+/CD163+ TAMs in microanatomical compartments of invasive tumor regions. No statistically significant trends were observed. Data for A–D were established by immunohistochemical staining of the tissue microarrays harboring 262 lung adenocarcinomas with antibodies for CD68 and CD163 followed by automated quantification of the stained cells and (for A and B) normalization against compartment area.

We next investigated whether there was an association between statin use and TAM number and polarity by quantitation of CD68/CD163 staining in statin users and nonusers among the 262 lung adenocarcinoma cohort. There was a marked dose-dependent trend towards reduced numbers of CD68+ luminal macrophages in areas of in situ tumor growth (Figure 4A), but this was not seen in areas of invasive growth (Figure 4B). With regard to macrophage polarity, increasing statin dosage showed a statistically significant association with diminished CD68+/CD163+ staining in parenchymal and stromal areas within in situ tumor regions (Figure 4C). Thus, within in situ areas, both the density of luminal macrophages and the degree of prooncogenic polarization are reduced with higher statin dosage, with the net result being that the absolute number of prooncogenic macrophages is reduced. This relationship, however, was absent in invasive regions (Figure 4D).

**Figure 5.**
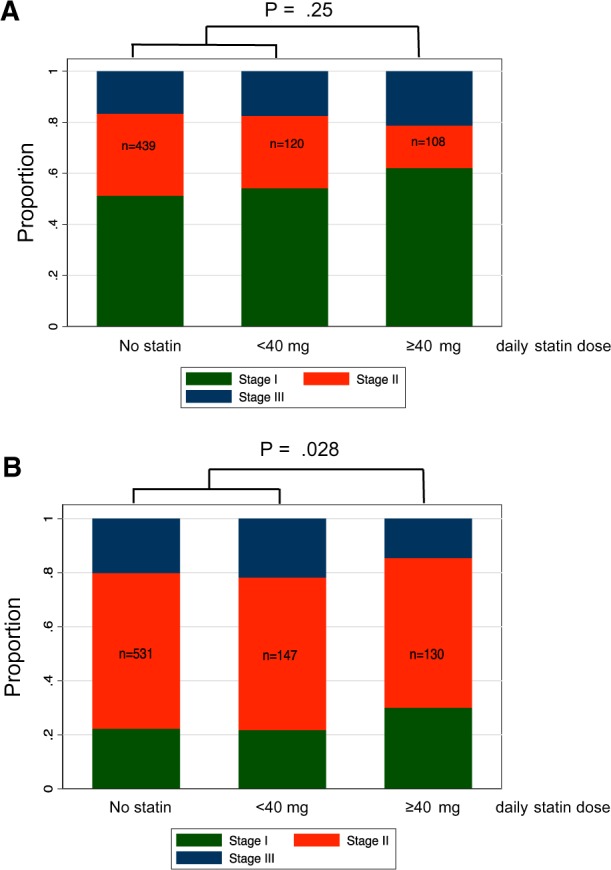
Relationship between statin use and lung adenocarcinoma stage and grade. **A**) Tumor stage in statin users and nonusers. Overall pathological tumor stage was determined and compared across the treatment groups. The **bar graph** indicates the proportion of tumors of each stage within patients receiving no statin, less than 40 mg/d statin, or greater than or equal to 40 mg/d statin. A pair wise test comparing all three stages among the greater than or equal to 40 mg/d statin users with low or nonstatin users is indicated. **B**) Predominant tumor growth pattern in statin users and nonusers. The predominant growth pattern was determined by inspection of whole H&E sections according to World Health Organization diagnostic criteria. Predominantly in situ tumors were classified as low risk, predominantly acinar or papillary cases as medium risk, and solid and micropapillary predominant cases as high risk. The **bar graph** indicates the proportion of tumors of each risk category receiving no statin, less than 40 mg/d statin, or greater than or equal to 40 mg/d statin. A pair wise test comparing all three risk groups among the greater than or equal to 40-mg/d statin users with low or nonstatin users is indicated.

To investigate if reduced CD68+/CD163+ TAMs within in situ regions has an impact on lung adenocarcinoma development, we first assessed the link between statin use and pathological tumor staging. Stage data were obtained for our entire tumor cohort of 958 patients, and information on statin use among this cohort is presented in Table 2. Although there appeared to be greater stage I tumors among the higher dose statin users, this trend was not statistically significant (Figure 5A).

**Table 2.** Statin use in entire lung adenocarcinoma cohort

Variable	No. (%)
Statin use	
No statin	538 (56.2)
Statin	282 (29.4)
Unknown	138 (14.4)
Statin type	
Atorvastatin	77 (27.3)
Simvastatin	177 (62.8)
Other	28 (9.9)
Statin dose	
<40 mg	152 (53.9)
≥40 mg	130 (46.1)
Statin duration	
<1 mo	94 (33.3)
1–6 mo	85 (30.1)
>6 mo	101 (35.8)
Unknown	2 (0.7)

A complication of correlating pathological stage with statin use is that stage at presentation is affected by multiple confounding factors, including the likely earlier presentation of statins users in the medical system and more regular monitoring than nonusers. We therefore investigated the relationship between statin use and tumor virulence (“grade”) by examining the predominant growth pattern, which is a measure of tumor virulence not known to be associated with clinical stage. Pulmonary adenocarcinomas fall into low-, medium-, and high-risk groups based on their predominant growth pattern (in situ vs acinar/papillary vs solid/micropapillary) (28–30). This classification is well established in the lung cancer pathology field and is likely to form the basis of a formal clinical grading system in the near future. Most lung adenocarcinomas exhibit several patterns of growth, and a minor proportion of in situ growth is common even in advanced cases; this can represent either a remaining part of the low-grade precursor lesion or an area of surface outgrowth of potentially invasive malignancy (31). Crucially, we found a statistically significant relationship between higher statin use and diminishing virulence as assessed by tumor growth pattern (Figure 5B), indicating that the transition to higher grade invasive growth patterns is delayed by statin activity.

**Figure 6.**
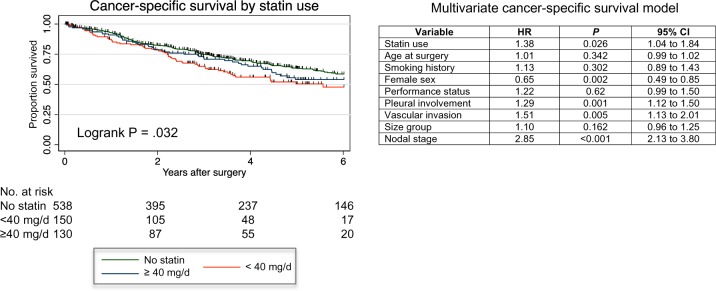
Relationship between statin use and cancer-specific survival. **Kaplan-Meier plot** showing the relationship between cancer-specific patient survival and statin therapy in the patient cohort. NB: two statin users were excluded from the survival model because they died on the day of surgery. The **table** on the right shows a multivariable Cox proportional hazards survival model as applied to these data. CI = confidence interval; HR = hazard ratio.

To determine if statin use has a long-term beneficial effect on outcomes for lung adenocarcinoma patients, we investigated the relationship with survival in this larger cohort but found that statin users had slightly worse cancer-specific survival compared with nonusers, although this relationship was not dose dependent (Figure 6). When incorporated into a multivariable Cox proportional hazards survival model, statin use remained an independent predictor of poor outcome (Figure 6), although the involvement of other comorbidities related to the prescription of statin such as cardiovascular disease and stroke cannot be excluded from this analysis.

## Discussion

There is no clear evidence for statins having either a beneficial or detrimental effect on cancer incidence or survival from cancer from epidemiological investigations (32). However, there has been much interest in investigating their potential as cancer preventive or therapeutic agents because they have powerful antiproliferative and antii-nflammatory properties within in vitro and in animal models (9,10). We have focused our attention on the anti-inflammatory roles of statins because macrophages are major mediators of inflammation and TAMs have both pro- and antitumorigenic functions (20,22). We reasoned that an impact on TAMs could potentially explain the controversial data linking statins with cancer imparted from epidemiological investigations.

Here, we provide evidence that statin use is associated with a reduction of protumorigenic TAMs within areas of in situ tumor growth in human lung adenocarcinomas (Figure 4C). The fact that this correlation is dose dependent reinforces this relationship. At present, it is not clear whether these effects are related to the ability of statins to inhibit the mevalonate pathway within TAMs with subsequent effects on macrophage polarity and tumor cell behavior, within tumor cells that then affects TAMs through paracrine mechanisms, or through some other mevalonate-independent function. Further investigations are required to unravel the precise mechanisms involved.

Stromal macrophages demonstrating M2 polarity are known to be associated with more aggressive disease in pulmonary adenocarcinoma (23–25), and, indeed, we have found that protumorigenic macrophage polarization correlates with tumor cell proliferation in primary tumor tissue (Figure 3A). Given published evidence for their oncogenic functions (20,24,25), it is logical to assume that lowering the density of M2-polarized TAMs within in situ regions by statins is likely to have the biological effect of slowing progression to invasive disease. Thus, potentially, through their association with diminished protumorigenic TAM polarity, statins can provide chemopreventive benefit. This idea is supported by the observed relationship between statin use and a shift towards a lower risk growth pattern (Figure 5B). However, a direct causal link between statin use, suppressed protumorigenic TAMs, and reduced growth pattern remains to be proven.

We find that the relationship between statin use and reduced protumorigenic TAMs is lost in invasive regions of human lung adenocarcinoma, suggesting that the dependency on paracrine signaling from TAMs is restricted to the in situ growth phase or perhaps that statin resistance emerges as the disease progresses. The reasons for this are not clear but may indicate alterations in the TAMs themselves or in the tumor-TAM interaction associated with transition to malignancy. It also suggests that any clinical value that statins may have in the treatment or prevention of lung adenocarcinoma may be most apparent very early in the natural history of the tumor, possibly years before malignancy becomes clinically apparent. Certainly, in our cohort of patients with established surgically treated disease, no overall benefit of statin use is seen. Additionally, any beneficial survival effects may have been partially masked by comorbidities associated with statin use such as cardiac disease.

In summary, our findings support a mechanistic role for statin drugs in the slowing or prevention of lung cancer progression via alterations in macrophage polarity. However, causal links between statin use, suppressed protumorigenic TAMs, and reduced tumor virulence remain to be proven mechanistically in appropriate model systems.

## Funding

This work was funded by a Ministry of Higher Education of Iraq scholarship to EAD, by MRC programme funding to JLQ, and by a CRUK program grant (C1362/A6969) awarded to CAP.

## Notes

The funders had no role in the design of the study; the collection, analysis, or interpretation of the data; the writing of the manuscript; or the decision to submit the manuscript for publication. There are no potential conflicts of interest to disclose.

## Notes

**Present address:** The University of Kufa, Department of Pathology and Forensic Medicine, Najaf, Iraq (EAJ).
